# The Long Non-coding RNA *Flatr* Anticipates Foxp3 Expression in Regulatory T Cells

**DOI:** 10.3389/fimmu.2018.01989

**Published:** 2018-09-25

**Authors:** Aleksandra Brajic, Dean Franckaert, Oliver Burton, Simon Bornschein, Anna L. Calvanese, Sofie Demeyer, Jan Cools, James Dooley, Susan Schlenner, Adrian Liston

**Affiliations:** ^1^Laboratory of Translational Immunology, VIB Center for Brain and Disease Research, VIB, Leuven, Belgium; ^2^Department of Microbiology and Immunology, University of Leuven, Leuven, Belgium; ^3^VIB Cancer Research Center, VIB, Leuven, Belgium

**Keywords:** Tregs, Foxp3, lncRNA, *Flatr*, iTreg induction

## Abstract

Mammalian genomes encode a plethora of long non-coding RNA (lncRNA). These transcripts are thought to regulate gene expression, influencing biological processes from development to pathology. Results from the few lncRNA that have been studied in the context of the immune system have highlighted potentially critical functions as network regulators. Here we explored the nature of the lncRNA transcriptome in regulatory T cells (Tregs), a subset of CD4^+^ T cells required to establish and maintain immunological self-tolerance. The identified Treg lncRNA transcriptome showed distinct differences from that of non-regulatory CD4^+^ T cells, with evidence of direct shaping of the lncRNA transcriptome by Foxp3, the master transcription factor driving the distinct mRNA profile of Tregs. Treg lncRNA changes were disproportionally reversed in the absence of Foxp3, with an enrichment for colocalisation with Foxp3 DNA binding sites, indicating a direct coordination of transcription by Foxp3 independent of the mRNA coordination function. We further identified a novel lncRNA *Flatr*, as a member of the core Treg lncRNA transcriptome. *Flatr* expression anticipates Foxp3 expression during *in vitro* Treg conversion, and *Flatr*-deficient mice show a mild delay in *in vitro* and peripheral Treg induction. These results implicate *Flatr* as part of the upstream cascade leading to Treg conversion, and may provide clues as to the nature of this process.

## Introduction

Sequencing of the human genome showed that there are only ~20,000 protein-coding genes, which is comparable to other less complex organisms such as nematodes or the fruit fly. This would suggest that eukaryotic genome must use other ways to generate biological complexity. Indeed, only 1–2% of the genome encodes protein sequences, with the other 98% potentially contributing to complexity through structural modification of accessibility and the generation of non-coding RNA molecules (ncRNA). One particular class of ncRNA, the long-non-coding RNA (lncRNA), have remained particularly enigmatic, long being dismissed as “transcriptional noise”. However, recent studies indicate that the ~35,000 mammalian lncRNA play a significant role in orchestrating and fine-tuning transcriptional programs both in health and disease (1–3). These lncRNAs can be located within the nucleus or the cytoplasm of a cell and may or may not be polyadenylated. LncRNAs can be categorized into groups according to their localization (intronic, intergenic) and transcriptional direction (sense, antisense, bidirectional) (1).

Very few functional studies of lncRNA have been performed, however the few in-depth studies published have revealed profound roles for these ncRNA. Within the haematopoeitic system, individual lncRNA regulate the survival of the myeloid lineage (4), with several known to be important for the differentiation of eosinophils (5), granulocytes (6), and dendritic cells (7). In addition to the involvement of lncRNA in differentiation, several studies describe their role in innate and adaptive immune response. The long intergenic non-coding RNA (lincRNA)-COX-2 plays a role in the activation or repression of immune-regulatory gene expression in macrophages (8). THRIL (TNF and heterogeneous nuclear ribonucleoprotein L related immunoregulatory lincRNA) is a key player regulating TNF-α transcription (9). PACER (p50-associated Cox2 extragenic RNA) is located upstream of the Cox2 transcriptional start site and helps the production of inflammatory mediators (10). NEAT1 (nuclear enriched abundant transcript 1 or nuclear paraspeckle assembly transcript 1) binds SFPQ (a paraspeckle protein) and prevents inhibition of IL-8 (11). Within T cells, ThymoD (thymocyte differentiation factor) aids thymic T cell development by inducing the expression of Bcl11b (12) while NeST enhances IFN-γ production in CD8^+^ T cells via and chromatin modification of the IFN-γ locus (13). Despite these important functions, it is notable that very few lncRNA have been identified via genetic screens, either due to intrinsic biases toward protein-coding genes in screening methods (14), or because (unlike the examples above) most lncRNA exert relatively subtle impacts on gene regulation, tweaking regulatory networks rather than controlling major phenotypes.

Regulatory T cells (Tregs) are a specialized subpopulation of CD4^+^ T cells which are critical for the maintenance of tolerance toward self (15). Dysfunction of these cells can result in severe autoimmune diseases, such as type 1 diabetes, multiple sclerosis, rheumatoid arthritis, colitis, and inflammatory bowel disease (16). However, a hyperactive function of these cells is also detrimental, as it can inhibit beneficial anti-pathogen (17) and anti-tumor immunity (18). Forkhead box P3 (Foxp3) is a key transcriptional regulator of Tregs and is used for their identification. Foxp3 is induced during the thymic selection process in a subset of CD4^+^ T cells with a certain degree of T cell receptor (TCR) self-reactivity, creating thymic Tregs (tTregs) (19). In addition to tTreg generation, Foxp3 induction can occur in T cells while they are present in other tissues, such the gut, colon or placenta, generating peripheral Tregs (pTregs) (20). Foxp3 expression results in radical transcriptional rewriting and functional differentiation, creating T cells with a largely suppressive phenotype (21). In mice and human, FOXP3-deficiency results in defective generation of functional Tregs and a fatal breach in immunological tolerance, causing highly aggressive multi-organ autoimmune pathology (22, 23).

Few studies have looked at the function of lncRNA in Tregs. In conventional T cells, expression of the lncRNA *lnc-EGFR* promotes the differentiation of Tregs, licensing tumor growth (24). In Tregs themselves, genomic deletion of the lncRNA *Flicr*, encoded within the *Foxp3* promoter region, results in altered expression of Foxp3 (25). While this latter study could arguably be due to the altered structure of the *Foxp3* promoter, owing to the cis-nature of the reported function, it strongly suggests that lncRNA will be of importance in controlling Treg transcriptional profiles. Here we systematically assess the lncRNA profile of Tregs, identifying a novel lncRNA that anticipates Foxp3 expression. This lncRNA, named here *Flatr* (Foxp3-specific lncRNA anticipatory of Tregs), is highly conserved and enriched in activated Tregs. Generation of Flatr-deficient mice resulted in a minor impairment of *in vitro* and peripheral Treg induction, indicating a biomarker, rather than major functional, role in the upstream cascade leading to Foxp3 expression.

## Results

### The Treg LncRNA transcriptome is shaped by Foxp3 expression

**Figure 1:**
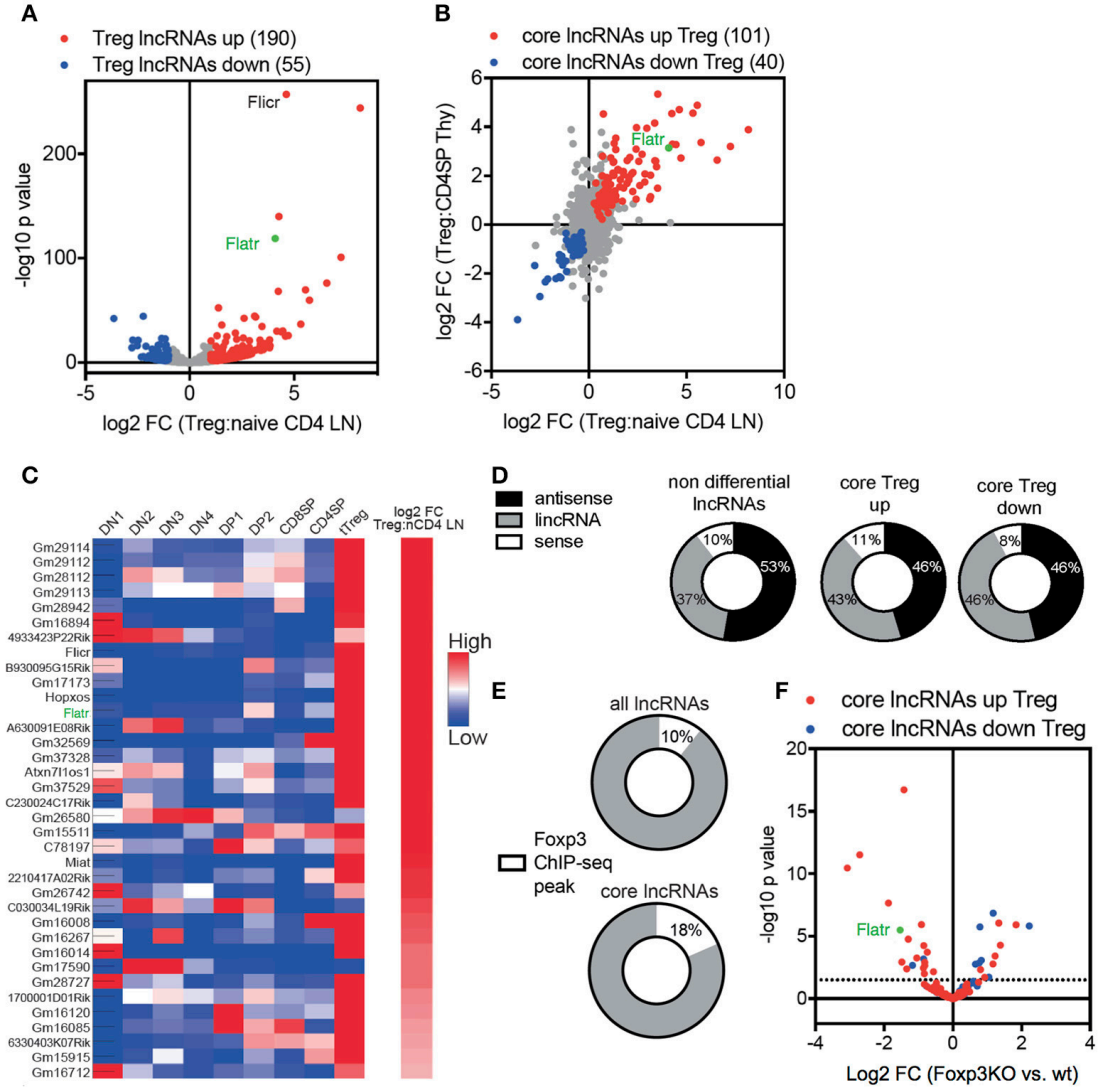
Treg lncRNA transcriptome is shaped by Foxp3 expression. **(A)** Volcano-plot showing differential expression of 1765 lncRNA in naïve CD4^+^ T cells (CD4^+^CD62L^+^CD44^−^GFP^−^) compared to Tregs (CD4^+^GFP^+^) from Foxp3GFP mice (*n* = 3 replicates from pooled biological samples). Flatr annotated and marked in green. Previously published Flicr annotated. Downregulated (blue). Upregulated (red). *P* < 0.05 cutoff for differential expression. **(B)** log2 fold change expression between naïve CD4^+^ T cells and Tregs, comparing thymic and peripheral subsets [(57), note only polyadenylated lncRNA present in database]. **(C)** Expression of selected, Treg-specific lncRNAs within thymic subsets of T cell development and peripheral naïve CD4^+^ T cells and Tregs [(57), note only polyadenylated lncRNA present in database]. **(D)** Non differential, core Treg upregulated, and core Treg downregulated lncRNAs grouped by genomic location relative to protein-coding genes [sense, antisense, and long intergenic non coding (lincRNA)]. **(E)** Foxp3 Chip-seq peaks (GSE40686) within the promoter region or the gene body of non-differential expressed lncRNAs or core Treg lncRNAs. **(F)** Differential expression of core lncRNAs in Foxp3^+^ Treg and Foxp3KIKO Treg (GSE40686).

In order to characterize the Treg-specific lncRNA transcriptome, we started with a high throughput sequencing approach. Foxp3^GFP^ reporter mice (26) were used as a source of naïve CD4^+^ T cells (CD4^+^CD62L^+^CD44^−^GFP^−^) and Treg (CD4^+^GFP^+^). To ensure efficient capture of all lncRNA, and not just those with a polyadenylated tail, we used ribosomal RNA (rRNA) depletion prior to Illumina HiSeq 2000 sequencing. Expression data was mapped onto known lncRNA, of which 1765 were expressed in Treg. Comparative expression analysis found that 13.8% of lncRNA expressed by Tregs were differentially expressed when compared to expression in naïve CD4^+^ T cells, with 190 lncRNA upregulated in Tregs and 55 lncRNA downregulated in Tregs (Figure 1A). Using published datasets, these lncRNA core signature changes in Tregs were reproducible and specific, with a tight correlation between thymic and peripheral expression (Figure 1B) and a similar Treg-specific expression pattern observed across different stages of T cell differentiation (Figure 1C).

Transcriptome analysis allowed us to define the sets of non-differential expressed lncRNA (i.e., expressed in both Treg and naïve CD4^+^ T cells, at equivalent levels), and the core Treg-signature lncRNA, with either upregulation or downregulation in Tregs. Comparison of the non-differential set to the core signature sets revealed three lines of evidence for active control of the lncRNA transcriptome in Tregs. First, core signature lncRNA, both up and down, were disproportionately likely to be intergenic in genomic position (Figure 1D), consistent with transcriptional control mechanisms independent of mRNA changes. Second, core signature lncRNA were twice as likely to possess a Foxp3 chromatin immunoprecipitation (ChIP) peak (27) within the transcript or promoter region (Figure 1E), suggesting a mechanism for direct Foxp3-guided expression modification. Third, when assessing the expression in Foxp3-deficient Tregs [using the *Foxp3*^*KIKO*^ transcriptome (28)], an enrichment was observed for the Treg upregulated lncRNA to be reduced in expression and the Treg downregulated lncRNA to be increased in expression (Figure 1F). Together, these results suggest that the unique lncRNA transcriptome in Tregs is, at least in part, shaped by Foxp3 expression.

### The LncRNA flatr is specifically expressed by a subset of activated tregs

**Figure 2:**
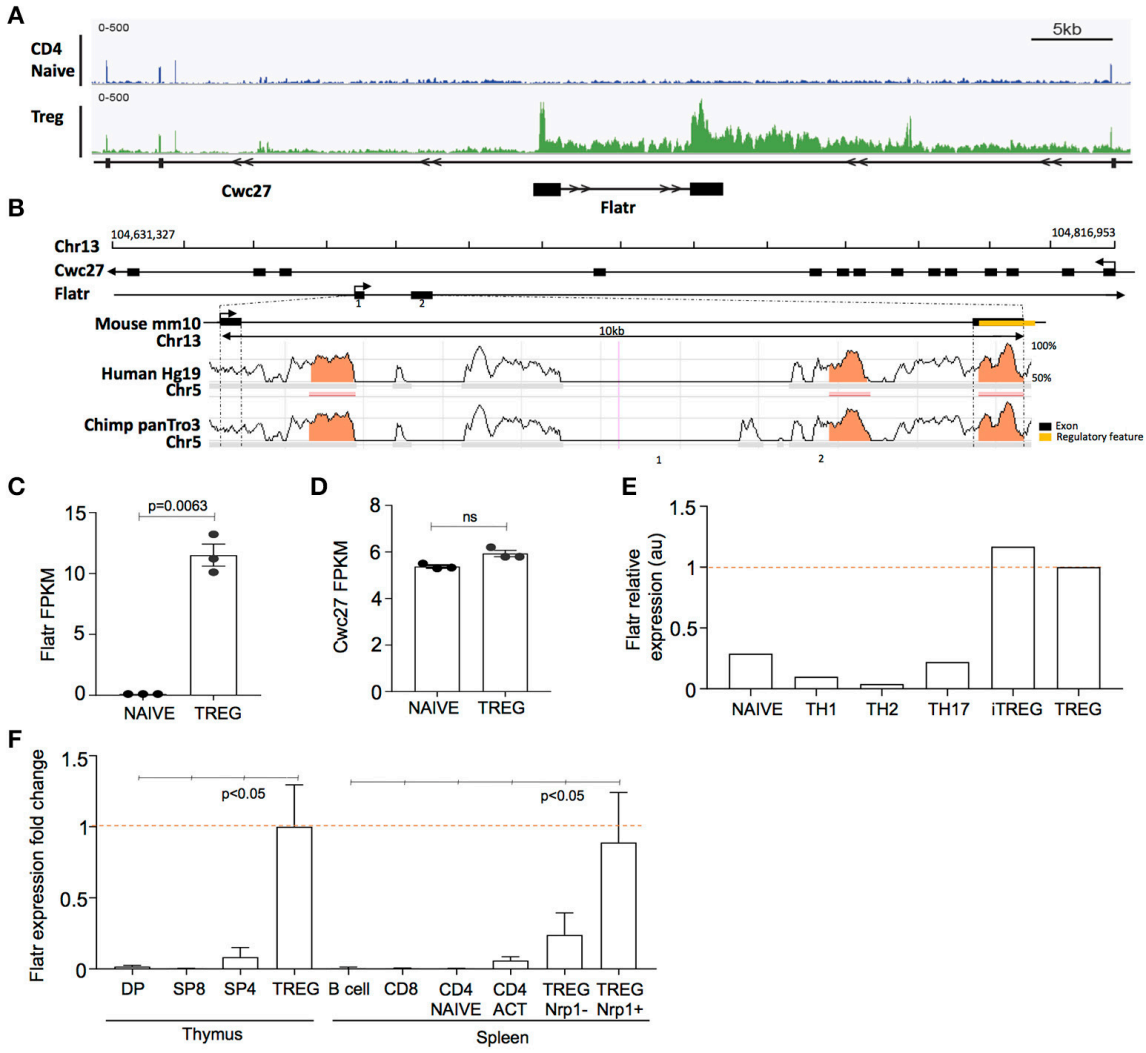
Flatr is a Tre-specific intronic lncRNA that anticipates Foxp3 expression. **(A)** Genomic plot showing location of murine Cwc27 with intronic location of Flatr and RNA-seq read data, visualized with IGV. **(B)** Sequence conservation across mouse, human, and chimpanzee. Regulatory region, annotated by Ensemble (ENSMUSR00000080103), in exon 2 is highlighted (yellow). Exons are marked by black bars. Regions of more than 100bp and with more than 70% similarity are marked (orange). **(C)** Flatr and **(D)** Cwc27 expression as FPKM values in naïve and Treg cells as determined by RNAseq analysis. Each point is an individual mouse. *N* = 3/group. FPKM, fragments per kilobase per million. **(E)** Flatr expression as determined in the T cell Th-express compendium (29). **(F)** RT-qPCR analysis on FACS purified populations showing Flatr expression relative to thymus Treg values. *N* = 6 per group. Data was normalized to RNA amount, Ppia and Rpl expression. Naïve CD4^+^ T cells (CD4^+^CD8^−^ CD62L^+^ CD44^−^); Treg, regulatory T cells (CD4^+^Foxp3^+^); DP, double positive CD4^+^CD8^+^ thymocytes; SP8, CD4^−^CD8^+^ thymocytes; SP4, CD4^+^CD8^−^ thymocytes; B cell (CD4^−^CD8^−^CD19^+^); CD8, CD8^+^ T cell; CD4 ACT, activated CD4^+^ T cells (CD4^+^CD8^−^CD44hiCD62Llo); Nrp1, Neuropilin.

Among the unique lncRNA transcriptome in Tregs, we selected *Flatr* for further study, as one of the most highly expressed Treg-specific lncRNA. *Flatr* is an intronic lncRNA, located within the gene *Cwc27* on chromosome 13 (Figure 2A). *Flatr* is highly conserved across species, with both exons showing >70% sequence similarity between mouse, human, and chimpanzee (Figure 2B). In our RNAseq experiment, *Flatr* expression was high in Tregs while essentially undetectable in naïve CD4^+^ T cells (Figure 2C). By contrast, the host gene, *Cwc27*, was unchanged between Tregs and naïve CD4^+^ T cells, indicating a specific alteration in the regulation of the lncRNA (Figure 2D). Analysis of the Th-express compendium (29) also indicated a Treg-specific expression pattern for *Flatr*, across different CD4^+^ T cell subtypes (Figure 2E). As an independent validation, we sorted lymphocyte populations from the thymus and spleen. *Flatr* expression was restricted to Tregs in both organs, and further showed 3-fold higher expression in Nrp1^+^ tTregs than Nrp1^−^ pTregs (Figure 2F).

**Figure 3:**
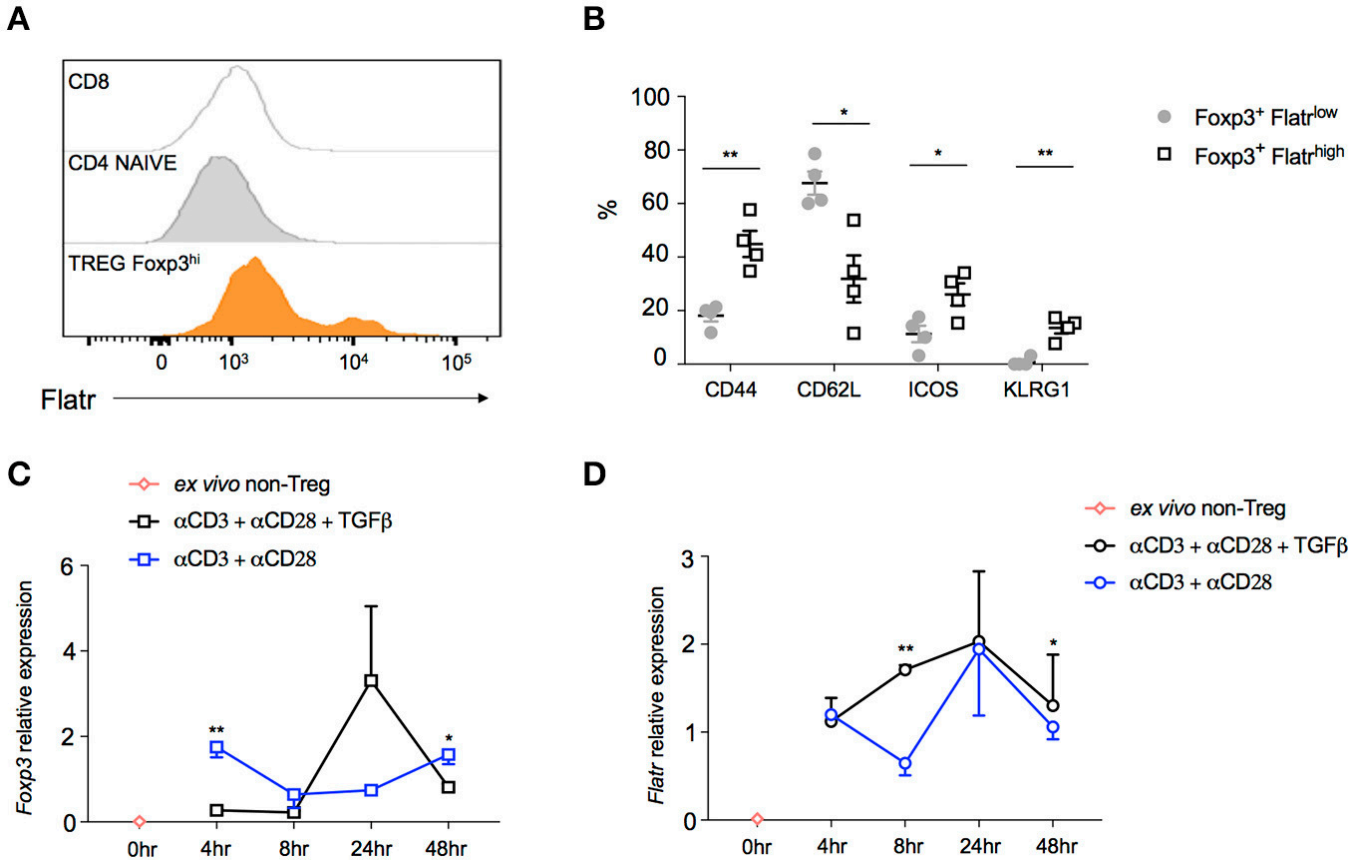
Characterization of Flatr-expressing Treg. **(A)** RNA Primeflow histogram showing expression of *Flatr* in different immune subsets, pooled from three mice. **(B)** Expression of activation markers in splenic *Flatr*^*low*^ Treg and *Flatr*^high^ Treg (*n* = 4). RT-qPCR analysis showing *Foxp3* **(C)** and *Flatr* **(D)** in cultured naïve T cells (CD4^+^ CD62L^+^ CD44^−^) from spleen and lymph nodes of wildtype mice activated in the presence of αCD3 and αCD28, with and without TGFβ at the indicated time points (*n* = 3–8). Normalized to *Ppia* and *Rpl* expression levels. All data are means ± SEM. *p < 0.05, **P < 0.01.

In order to determine whether *Flatr* was expressed by all Tregs, or by only a subset, we adapted RNAflow to the detection of lncRNA, a procedure complicated by the relative shortness of lncRNA limiting RNAflow probe sites. The resulting RNAflow profiles both confirmed the Treg-specificity of Flatr expression, and demonstrated that only a subset of Treg express *Flatr* at high levels (Figure 3A). Phenotyping analysis of Flatr^low^ vs. Flatr^high^ Tregs indicated that Flatr^high^ Tregs were more activated, with a shift toward being CD62LlowCD44hi and also elevated expression of ICOS and KLRG1 (Figure 3B). When analysing the expression of *Foxp3* and *Flatr* during Treg induction *in vitro*, Foxp3 induction is not efficiently induced until 24 h post-stimulation, and is dependent upon TGFβ (Figure 3C). By contrast, *Flatr* expression precedes *Foxp3* expression, with substantial upregulation from 4 h post-stimulation, and was only partially TGFβ-dependent (Figure 3D). Together, these results demonstrate that *Flatr* is a Treg-specific lncRNA, with expression enriched within the subset of activated Tregs.

### Delayed *in vitro* and peripheral treg induction in flatr knockout mice

**Figure 4:**
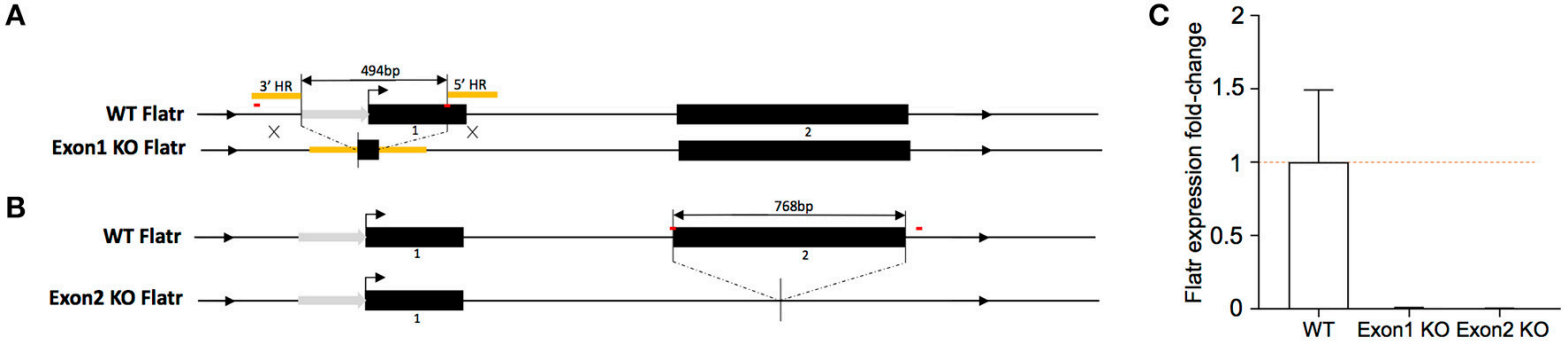
Generation of Flatr deficient mice. **(A)** Schematic depiction of *Flatr* locus on Chr13 and targeting strategy for exon 1 KO mice with CrispR-Cas9 mediated homologous recombination deleting 494bp containing the majority of exon 1 and ca. 230bp upstream region. HR, homologous region (yellow). Putative promoter region indicated with gray arrow. CrispR binding sites are marked in red. **(B)** Schematic depiction of the *Flatr* locus on Chr13 and targeting strategy for exon 2 with CrispR-Cas9 mediated donor-free deletion of 768bp containing exon 2, 4bp upstream and 131 downstream sequence. Promoter region indicated with gray arrow. CrispR binding sites are marked in red. **(C)** RT-qPCR analysis showing *Flatr Foxp3* expression levels in WT, Exon1KO and Exon2KO FACS-purified Treg cells relative to WT levels (*n* = 6). Normalized to *Ppia* and *Rpl* expression levels.

To test the function of *Flatr* in Tregs, we generated new knockout mice deficient in the lncRNA. Two independent strains were generated. In the first, “exon 1 KO mice”, CrispR-Cas9-mediated homologous recombination deleted the promoter region and the majority of exon 1 (Figure 4A). In the second, “exon 2 KO mice”, donor-free CrispR-Cas9 deletion was used to remove the entirety of exon 2 (Figure 4B). In both mouse strains, sorted Tregs demonstrated a complete absence of *Flatr* transcript (Figure 4C), validating the models for analysis of *Flatr* deficiency.

**Figure 5:**
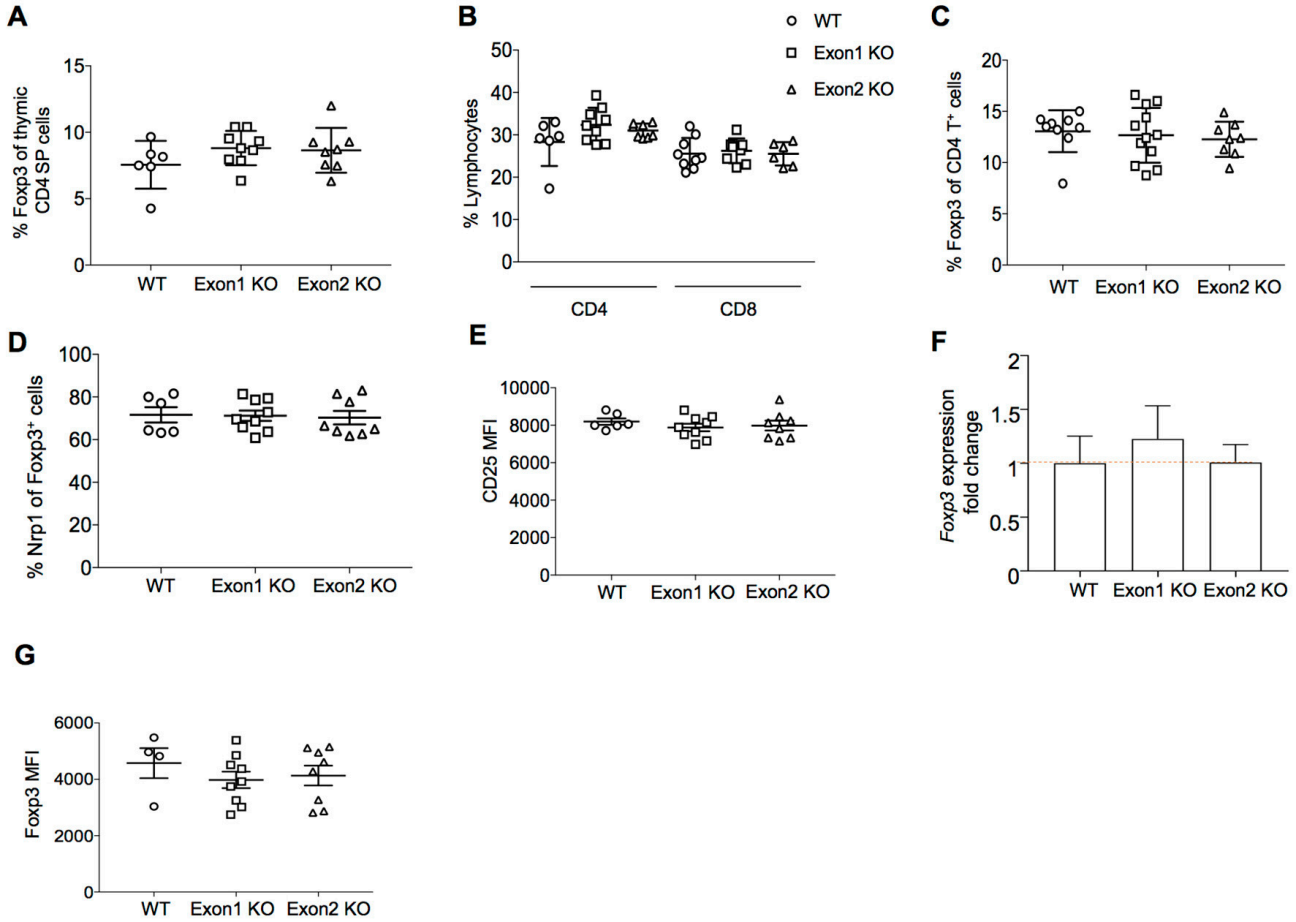
*Flatr* deficient T cells show normal thymic Treg induction. **(A)** Percentage of CD4 thymic SP Foxp3^+^ Treg cells from wild-type (WT), Exon 1 KO and Exon 2 KO mice (*n* = 6–9). **(B)** Percentage of CD4^+^ and CD8^+^ cells within total lymphocytes from lymph nodes (LN) of WT, Exon 1 KO, and Exon 2 KO mice (*n* = 6–9). **(C)** Percentage of CD4^+^ Foxp3^+^ Treg cells in LN from WT, Exon 1 KO, and Exon 2 KO mice (*n* = 8–12). **(D)** Percentage of Nrp1^+^ Foxp3^+^ Treg cells in LN from WT, Exon 1 KO, and Exon 2 KO mice (*n* = 6–9). **(E)** CD25 (MFI) in the CD25^+^ Treg cell population from LN of WT, Exon 1 KO, and Exon 2 KO mice (*n* = 6–9). **(F)** RT-qPCR analysis showing *Foxp3* expression levels in splenocytes from WT, Exon 1 KO, and Exon 2 KO mice relative to WT levels (*n* = 3). Normalized to *Ppia* and *Rpl* expression levels. **(G)** Foxp3 mean fluorescence intensity (MFI) in the Foxp3^+^ Treg cell population from LN of WT, Exon 1 KO, and Exon 2 KO mice (*n* = 4–9).

A potential role for *Flatr* in Treg development was assessed through analysis of Exon 1 KO and Exon 2 KO mice. We first assessed the role of *Flatr* in thymic Treg development *in vivo*. Both strains showed normal T cell development in the thymus (data not shown), with no alteration in the production rate of Tregs in the thymus (Figure 5A). In the periphery, the relative proportions of CD4 and CD8 T cells were unchanged (Figure 5B). The percentage of Tregs within the CD4^+^ T cell compartment remained intact (Figure 5C), with no alterations in the balance of thymic- and peripherally-derived Tregs (Figure 5D), indicating no essential role for *Flatr* in Treg differentiation or development. Consistent with this, expression of CD25, the high affinity receptor required for IL-2-mediated Treg homeostasis (30) remained unchanged on Tregs from KO mice (Figure 5E). Unlike *Flicr* (25), encoded in the Foxp3 promoter, deletion of *Flatr* did not alter the expression of Foxp3 at either the mRNA (Figure 5F) or protein (Figure 5G) level.

**Figure 6:**
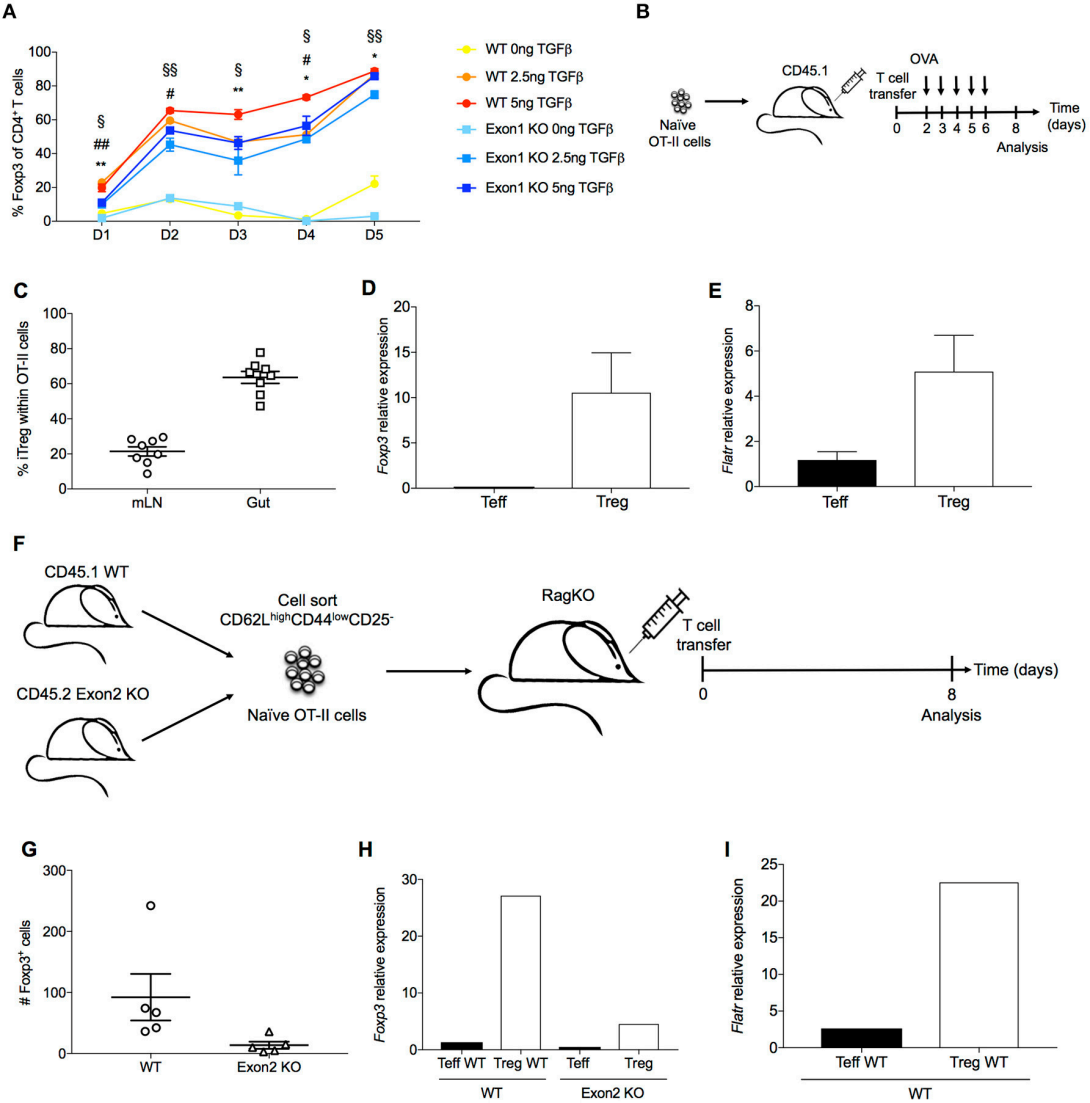
*Flatr* deficient T cells show delayed Treg induction *ex vivo* and *in vivo*. **(A)** Cultured naïve T cells (CD4^+^ CD62L^+^ CD44^−^) from spleen and lymph nodes of wildtype mice and Exon1 KO mice were activated in the presence of IL-2, αCD3, and αCD28, with and without TGFβ at the indicated time points (*n* = 3, representative of 8 experiments). *P < 0.05, **P < 0.01 for WT vs Exon1 KO at concentration of 0ng of TGFβ; #*P* < 0.05, ##*P* < 0.01 for WT vs. Exon1 KO at concentration of 2.5 ng of TGFβ; §*P* < 0.05, §§*P* < 0.01 for WT vs. Exon1 KO at concentration of 5ng of TGFβ. All data are means ± SEM. **(B)** Experimental design for oral antigen-induced Treg generation. 10^6^ naïve OT-II cells were transferred to a CD45.1 host. Mice were gavaged with OVA until analysis. **(C)** Frequency of Foxp3^+^ cells among transferred OT-II T cells (*n* = 4). **(D)** *Foxp3* mRNA levels in OT-II iTreg (*n* = 4). **(E)** *Flatr* expression in OT-II iTreg (*n* = 4). **(F)** Experimental design for Treg induction through homeostatic expansion. Naïve CD4 T cells from both wildtype (CD45.1) and Exon1-deficient mice (CD45.2) were transferred into a Rag-deficient host, and assessed after homeostatic expansion. **(G)** Flow cytometric quantification of Treg number in host mice, within the wildtype (CD45.1) and Exon1KO (CD45.2) compartments. **(H)** CD45.1 and CD45.2 CD4 Teff and Treg cells were sorted from homeostatic expansion mice, with RT-qPCR analysis of *Foxp3* and **(I)** *Flatr* (*n* = 4–8). Expression was normalized to *Ppia* and *Rpl* expression levels. All data are means ± SEM.

As *Flatr* expression anticipated *Foxp3* expression during *in vitro* induction (Figure 3D), we next assessed a functional role for Flatr in this process. Using a TGFβ-dependent Treg induction assay, we compared the ability of wildtype and Exon1KO naïve T cells to convert to the Treg lineage. Wildtype naïve T cells demonstrated progressive conversion to the Foxp3^+^ Treg lineage, a process which demonstrated a TGFβ dose-response (Figure 6A). Exon1KO naïve T cells exhibited a small but reproducible defect in this Treg conversion process (Figure 6A). The defect was present across the limiting TGFβ range and across the conversion period (Figure 6A), consistent with a general dampening of Treg conversion. Similar results were observed in culture conditions where IL-2 was limited, although under these conditions wildtype Tregs showed an expansion-contraction kinetics (Supplementary Figure 1). We further investigated peripheral Treg induction *in vivo*, using oral tolerance and homeostatic conversion assays. OVA-reactive OT-II naïve T cells were transferred into a congenic host and gavaged with OVA (Figure 6B). Using this system, pTreg induction is observed within the mesenteric lymph nodes and the gut (Figure 6C). These pTregs expressed both *Foxp3* (Figure 6D) and *Flatr* (Figure 6E), demonstrating Flatr upregulation during pTreg induction. To assess a function role during pTreg induction we switched to a homeostatic expansion system. Wildtype and Exon2 KO naïve T cells were injected into a Rag-deficient host (Figure 6F). This system results in pTreg conversion (Figure 6G), with upregulation of both *Foxp3* (Figure 6H) and *Flatr* (Figure 6I). Using this system, Flatr-deficient naïve T cells demonstrated a lower pTreg induction (Figure 6G) and reduced expression of Foxp3 in the converted Tregs (Figure 6H). Together, these results demonstrate a small but reproducible impact of Flatr expression in Treg induction both *in vitro* and *in vivo*.

**Figure 7:**
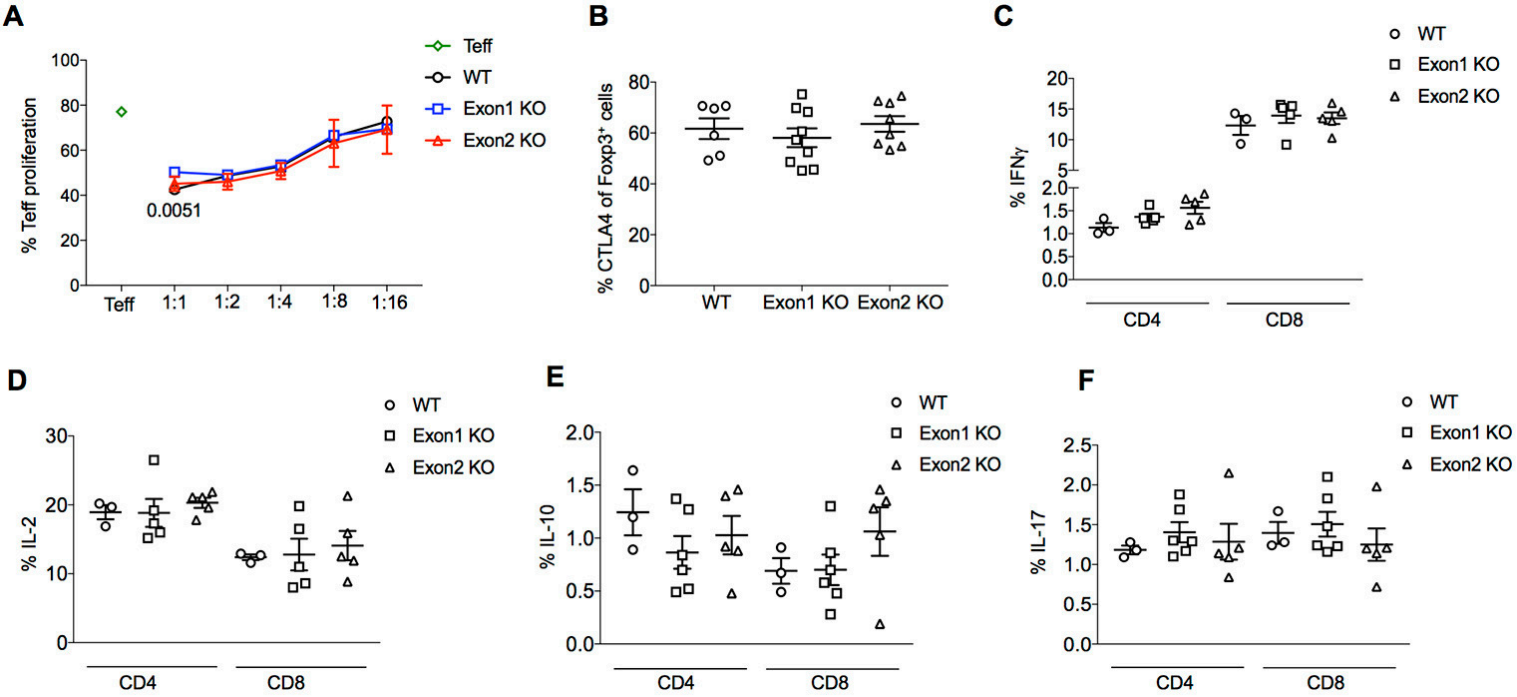
*Flatr* deficient Tregs retain normal function. **(A)** *in vitro* suppression assay data for the proliferation of naïve T cells (CD4^+^ CD62L^+^ CD44^−^) following the addition of Foxp3^+^ Tregs purified from the spleen and lymph nodes from WT and KO mice. Proliferation at different naive/Treg ratios shown (*n* = 6). The *p*-value in 1:1 ratio refers to Exon1 KO versus WT. **(B)** Percentage of lymph node Foxp3^+^ Treg cells expressing CTLA4. Data collected by flow cytometry from wildtype, Exon 1 KO and Exon 2 KO mice (*n* = 6–9). **(C–F)** IFNγ, IL-2, IL-10, and IL-17 production by CD4^+^ and CD8^+^ T cells from WT, Exon 1 KO and Exon 2 KO mice (*n* = 3–6). All data are means ± SEM. *p < 0.05.

No substantial difference was observed in Treg suppressive function. *In vitro* suppression of effector T cells was normal when comparing Tregs from KO mice to those from wildtype mice, with no major changes in the proliferation rate of cocultured naïve T cells following stimulation (Figure 7A). In an *in vivo* setting, the expression of the major suppressive mediator, CTLA4, was normal (Figure 7B). A global transcriptional analysis, performed by RNAseq experiments on purified CD4^+^CD25^+^ Tregs from wildtype, Exon 1 KO and Exon 2 KO mice, showed tight clustering of Tregs from each genotype, indicating that *Flatr*-deficiency does not dramatically alter the Treg transcriptional profile (Supplementary Figure 2). Analysis of differentially expressed genes found few transcriptional changes – only 36 significantly upregulated genes and 8 significantly downregulated genes were consistent across the two knockout strains when compared to wildtype cells (Supplementary Worksheet 1), with no significantly enriched pathways or known suppressive mediators. A normal suppressive capacity was borne out by *ex vivo* analysis of effector T cells in Flatr-deficient mice, with no spontaneous increase in effector T cells was observed (Figures 7C–F). These results identify *Flatr* as an anticipatory lncRNA during Treg induction without substantially impacting Treg suppressive capacity under homeostatic conditions.

## Discussion

The stable suppressive phenotype of Tregs is induced and maintained through the activity of the master transcription factor Foxp3 (31, 32). Foxp3 is well-documented to act as a transcriptional regulator of mRNA. Foxp3 binds with multiple protein partners (33, 34), which can in turn bind to ~700 gene targets (21) and either enhance or suppress the expression of the resulting mRNA, depending on the composition of the complex (34). Several of these targets include secondary transcription factors which serve to “lock in” the Foxp3-induced mRNA transcriptome (35), providing redundancy to the transcriptional circuit. Beyond the abundant data that Foxp3 drives the Treg fate through classical transcriptional control, there is growing evidence that Foxp3 also initiates non-classical programs that influence the stability and function of the lineage. At the DNA level, Tregs undergo extensive epigenetic reprogramming that stabilizes cell identity (36). This is particularly relevant to the *Foxp3* locus itself, which undergoes specific DNA demethylation and histone modifications in Tregs (37–39), however extensive modifications occur at a genome-wide level in functionally relevant genes (40). At the RNA level, Foxp3 not only initiates a mRNA transcriptional program, but also a unique microRNA signature (41). Loss of the Treg microRNA transcriptome results in a loss of suppressor phenotype during inflammation, resulting in fatal autoimmunity (42–44). These effects are partially mediated through miR-146a (45), miR-155 (46), and miR-17 (47), and are heavily intertwined with the transcriptional control initiated by Foxp3. Our study, and recent advances, implicate lncRNA transcription as an additional layer of complex cellular regulation initiated by Foxp3 and relevant to the Treg fate.

The impact of lncRNA on cellular biology can occur at the epigenetic, transcriptional and post-transcriptional level. There is growing evidence that lncRNA are involved in Treg biology. *MEG3*, a lncRNA expressed in CD4^+^ T cells, contributes to the imbalance of Tregs/Th17 ratio in patients with immune thrombocytopenic purpura (48). Expression of lncRNA *DQ786243* in Jurkat cells modulates Foxp3 expression, indicating a potential *in vivo* function (49). The lnc-epidermal growth factor receptor (*lnc-EGFR*) is highly expressed in Tregs and activates the expression of EGFR and its downstream AP-1/NF-AT1 axis, which increases Treg immunosuppressive function (24). The lncRNA *Flicr*, negatively regulates Foxp3 expression in Tregs through modification of chromatin accessibility, and alters the course of autoimmune diabetes (25). Here we have defined the scope and nature of the lncRNA transcriptome in Tregs, and identified *Flatr* as a biologically relevant lncRNA that anticipates Foxp3 expression during Treg differentiation and plays a functional role in the *in vitro* and peripheral conversion process.

The induction of Tregs from thymocytes or naïve T cells is a coordinated process involving TCR stimulation and costimulatory signals, which, depending on the context, can include signaling from IL-2, TGFβ, and CD28, among others. These upstream signals leave a permanent signature on the nascent Treg, indeed, part of the “Treg” signature is independent of Foxp3 itself, and instead driven by these upstream coordinators (50). Our results indicate that *Flatr* expression is part of this upstream induction process, as expression of *Flatr* precedes that of *Foxp3* itself. Nevertheless, after initial expression, Flatr becomes dependent on Foxp3 for continued high level expression, as it is reduced in Foxp3^KO^ “Tregs”, a pattern found in several other Treg signature genes, such as CD25 (51). These signals can be differentially integrated by the Foxp3 locus, which contains three conserved non-coding sequences (CNS) that regulate Foxp3 expression (39). Our data on *Flatr* indicates both a bias toward activated Tregs. Expression of *Flatr* is largely driven through αCD3/αCD28 stimulation, and accentuated by TGFβ. Consistent with this, *in vitro* and *in vivo* models of Treg induction that are dependent on strong TCR signal demonstrated a (minor) function role of *Flatr* in the process. CSN1 is involved in peripheral Treg induction (39), with TGFβ working via a Smad3 binding site in the region (52). CNS2 aids heritable activation of the Foxp3 locus (53, 54), with TGFβ signaling participating indirectly through Stat5 activation (55). A role in either, or both, of these processes is possible for *Flatr*, based on the induction data. Both of these elements are less potent than CNS3, which has critical importance for thymic induction, and appears to be TGFβ-independent (39). By contrast, *Flicr* is thought to work via CNS3, which potentially accounts for the more prominent, although still minor, phenotype (25). Like most non-coding RNA, the effect of *Flatr* and *Flicr* are subtle, likely to modulate these processes rather than acting as a qualitative “on-off” switch. Nonetheless, the conservation of these lncRNA suggests that such modulatory effects, while biologically silent under homeostatic conditions, are likely to manifest with altered pathology under stress conditions.

## Materials and methods

### Mice

Foxp3GFP mice (26) were used for lncRNA profiling. Two independent lines of Flatr KO mice were generated, targeting exon 1 and exon 2 of the Flatr transcript, respectively. C57BL/6 zygotes were injected with CrispR-Cas9 conjugates with gRNA flanking the targeted exon. Resulting offspring were screened for deletions, crossed to C57BL/6 mice and intercrossed to generate experimental mice. Flatr KO mice were backcrossed to Foxp3^Thy1.1^ mice for cell sorting (56). Antigen presenting cells were collected from Rag1 KO mice on the B6 background. Transfer experiment included Rag1 KO on the B6 background. OT-II OVA-specific TCR-transgenic mice on a C57BL6 background were obtained from The Jackson Laboratory (Bar Harbor, ME). This study was carried out in accordance with the recommendations of the European Union (EU) concerning the welfare of laboratory animals as declared in Directive 2010/63/EU and University of Leuven ethics committee. The protocol was approved by University of Leuven ethics committee.

### LncRNA sequencing and analysis

Tregs (CD4^+^ GFP^+^) and naïve T cells (CD4^+^ CD62L^+^ CD44^−^ GFP^−^) from *Foxp3*^*GFP*^ reporter mice (26) were sorted by FACS. RNA was isolated using NucleoSpin RNA isolation kit (Macherey-Nagel). RNA concentration and purity were determined spectrophotometrically using the Nanodrop ND-1000 (Nanodrop Technologies) and RNA integrity was assessed using a Bioanalyser 2100 (Agilent). Per sample, 400 ng of total RNA was used as input. Using the Illumina TruSeq® Stranded Total RNA Sample Prep Kit with Ribo-Zero Gold (protocol version “April 2013”) rRNA was depleted from the total RNA samples using Ribo-Zero ribosomal RNA reduction chemistry. Subsequently, RNA was purified and fragmented and converted into first strand cDNA in a reverse transcription reaction using random primers. Next, double-stranded cDNA was generated in a second strand cDNA synthesis reaction using DNA Polymerase I and RNAse H. The cDNA fragments were extended with a single “A” base to the 3′ ends of the blunt-ended cDNA fragments after which multiple indexing adapters were ligated introducing different barcodes for each sample. Finally an enrichment PCR was carried out to enrich those DNA fragments that have adapter molecules on both ends and to amplify the amount of DNA in the library. Sequence-libraries of each sample were equimolarly pooled and sequenced on 1 lane of a HiSeq2000 flow-cell at 2 × 100 bp. RNA-sequencing data was first cleaned (i.e., removal of adapters and low quality parts) with the fastq-mcf software after which a quality control was performed with FastQC. The reads were then mapped to the *Mus musculus* (mm10) genome with Tophat2. To identify the gene expression HTSeq-count was used to count the number of reads per gene. These read count numbers were then normalized to the sample size. Differential gene expression analysis was performed with the R-package DESeq2. RNA-seq data from mouse thymic T cell subsets (GSE48138) were used for the analysis of lncRNAs expression in thymocytes (57). The GSE40686 dataset was used to analyze the expression of lncRNAs in wildtype and *Foxp3*^*KIKO*^ Treg, as well as for Foxp3 Chip-seq dataset (27).

### RNAseq analysis of flatr-deficient cells

Tregs (CD4^+^ Thy1.1^+^) were sorted from *Foxp3*^*Thy*1.1^ Exon 1 KO, Exon 2 KO, and WT mice by FACS. Total RNA was isolated using RNeasy Mini kit (Qiagen). RNA concentration and purity were determined spectrophotometrically using the Nanodrop ND-1000 (Nanodrop Technologies) and RNA integrity was assessed using a Bioanalyser 2100 (Agilent). 3′mRNA-seq library preparation and transcriptome analysis was performed by Lexogen (Austria) using the QuantSeq 3′mRNA-Seq Library Prep Kit for Illumina and QuantSeq data analysis workflow.

### Locus mapping and analysis

The *Flatr* 3′ terminus was mapped using RNA sequencing of the identical dataset as *Foxp3*^*GFP*^ FACS-sorted Treg RNA sequencing expression analysis and visualized using IGV browser. To map the 5′ terminus, RNA was prepared from FACS-sorted Treg from *Foxp3*^*Thy*1.1^ mice (56) and cells were lysed and stored in TRIzol and RNA was extracted followed by phenol-chloroform extraction. 5′ RACE was performed using the RLM-RACE kit (ThermoFisher) as per manufacter's instructions, followed by subcloning using the CloneJET PCR cloning kit (ThermoFisher) and sanger sequencing. The 5′ prime terminus mapping was confirmed by analysis of FANTOM(5) CAGE data (58). Analysis of evolutionary conserved regions within the *Flatr* locus was assessed and visualized using ECR browser (59).

### Expression analysis

T cell subsets were FACS sorted from Foxp3^Thy1.1^ mice (56) and were found to be over 98% pure at the post-sort purity check. Cells were lysed and stored in TRIzol and RNA was extracted followed by phenol-chloroform extraction. cDNA was prepared using the GoScript Reverse transcription system (Promega) as per manufacturer's protocol. RNA-amount were normalized to cell number prior to cDNA generation. *Flatr* cDNA was amplified using forward primer (5′-ACTGGGACCATGAAAGTGCT-3′) and reverse primer (5′-TCCTGGCTCAGCAGTGATCT-3′) using the SYBR Green Real-Time PCR Master mix (ThermoFisher). Expression data was analyzed using the 2^−ΔΔ*CT*^ method and normalized to expression levels of *Rpl* and *Ppia*.

### Flow cytometry and RNA flow

Single-cell suspensions were prepared from mouse spleens. For intracellular cytokine staining, lymphocytes were plated at 1 × 106 cells/well in 96-well tissue-culture plates in complete RPMI containing phorbol 12-myristate 13-acetate (50 ng/mL; Sigma-Aldrich), ionomycin (250 ng/mL, Sigma-Aldrich), and monensin (1:1,500; BD Bioscience, San Jose, Calif) for 4 h at 37°C. All cells were fixed and permeabilized with the eBioscience Foxp3 staining kit (eBioscience). Mice were analyzed using the following antibodies: anti-CD3 (17A2), anti-CD4 (GK 1.5), anti-CD8 (53-6.7), anti-NK1.1 (PK136), anti-CD44 (IM7), anti-CD45.1 (A20), anti-CD45.2 (104), anti-CD25 (PC61.5), anti-CD62L (MEL-14), andti-ICOS (C398.4A), anti-KLRG1 (2F1.KLRG1), anti-cytotoxic T lymphocyte–associated antigen 4 (CTLA4; UC10-4B9), anti-Neuropilin 1 (3DS304M), anti-Foxp3 (FJK-16s), anti-Thy1.1 (HIS51), anti-IL-2 (JES6-5H4), anti-IL-10 (JES5-16E3), anti-IL-17 (SCPL1362), anti-IFN-γ (XMG1.2), and fixable Viability Dye eFluor™ 780 from eBioscience, Biolegend, BD bioscience, and Abcam. For RNA staining analyses, cells were treated according to the manufacturer's instructions using a commercially available kit (PrimeFlow RNA Assay, ThermoFisher) and compatible commercially available probes for β-actin (used as a positive control for RNA staining) and Foxp3 and specifically designed probe for *Flatr* (ThermoFisher). Data were collected on a BD FACSCanto II and BD FACSymphony (BD Biosciences) and analyzed with FlowJo software for Mac, version 10 (TreeStar, Ashland, Ore).

### *In vitro* suppression assay

Tregs (CD4^+^ CD25^+^ CD44^+^) and conventional T cells (CD4^+^ CD62L^+^ CD44^−^) from spleen and lymph nodes were purified using MagniSort-CD4^+^ T-cell enrichment kit (eBioscience) according to the manufacturer's instructions. Purified cells were FACS sorted and were found to be over 98% pure at the post-sort purity check. CD4^+^ conventional T cells (1 × 10^5^) were labeled with CellTrace Violet (ThremoFisher Scientific) according to the manufacturer's instructions and cultured in U-bottom 96-well plates for 5 days with Tregs at ratios Tregs:Tconv (1:1, 1:2, 1:4, 1:8, and 1:16) in the presence of RagKO splenocytes (5 × 10^4^) and 0.25 μg/ml of anti-CD3 (145-2C11) antibody (eBioscience). After 5 days cells were stained with fixable viability dye, CD4, and Foxp3 and data was collected using BD FACSCanto II (BD Bioscience). Data was analyzed with FlowJo software for Mac, version 10 (TreeStar, Ashland, Ore). The percentage of proliferating Tconv cells was determined by CellTrace Violet dilution and unlabeled CellTrace Violet-negative Tregs were excluded.

### *In vitro* induction assay

Naïve T cells (CD4^+^ CD62L^+^ CD44^−^) from spleen and LN were purified using MagniSort-CD4^+^ T-cell enrichment kit (eBioscience) according to the manufacturer's instructions after which the purified cells were FACS sorted and were found to be over 98% pure at the post-sort purity check. Sorted cells were seeded in 24 (2.5 × 10^5^) and 96-well plate (1 × 10^5^) precoated overnight with 2 μg/ml of anti-CD3 (145-2C11) and 5 μg/ml of anti-CD28 antibodies (37.51) (eBioscience) in complete RPMI in presence of TGFβ (dose titrated, R&D systems), and IL-2 (0 or 10 ng/ml; eBioscience) for stimulation. Cells were cultured up to 5 days and collected at different time points for the flow cytometry analysis of Treg induction and expression analysis of *Flatr* and *Foxp3* gene by Real time PCR.

### *In vivo* iTreg assays

Naïve T cells from spleen and LN of OT-II mice were enriched via negative selection utilizing MACS to deplete cells expressing CD25, CD19, CD11b, CD11c, NK1.1, F4/80, Ly-6G, CD8α, and Ter119 on MACS LS columns with anti-biotin and anti-CD44 microbeads (Miltenyi Biotec). Purified cells were found to be over 98% pure. Cells (1 × 10^6^) were injected i.v. into CD45.1 Foxp3^Thy1.1^ mice. Recipients were gavaged with OVA grade II (70 mg; Sigma-Aldrich) in 200 μl PBS from day 2 until day 6. On day 8 cells were collected from mesenteric lymph nodes and the gut and analyzed via flow cytometry. Additionally, cells from mesenteric lymph nodes were sorted (CD4^+^CD45.1^−^CD25^+^GITR^+^ and CD25^−^ cells from OT-II population and CD4^+^ CD45.1^+^Thy1.1^+^ and Thy1.1^−^ cells from host population) and analyzed for Treg induction by qPCR.

In Rag^KO^ transfer experiment, naïve T cells (CD4^+^CD62L^+^CD44^−^) from spleen and LN from spleen and lymph nodes of CD45.1 Foxp3^Thy1.1^ mice and CD45.2 Foxp3^Thy1.1^ Exon2 KO mice were purified using MagniSort-CD4^+^ T-cell enrichment kit (eBioscience) according to the manufacturer's instructions. Purified cells were FACS sorted and were found to be over 98% pure at the post-sort purity check. Naïve T cells from CD45.1 WT and CD45.2 Exon2 KO mice were mixed at a 1:1 ratio and injected (2 × 10^6^) i.v. into Rag^KO^ mice. After 8 days mice were sacrificed and cells isolated from spleen and lymph nodes were sorted and analyzed for Treg induction by qPCR and flow cytometry.

### Statistics

Statistical analyses were performed with Prism software (GraphPad Software) using the Student *t*-test. Error bars represent SEM as indicated. *p* < 0.05 was considered statistically significant.

## Author contributions

AB, DF, OB and AC performed the experiments. DF and SB performed bioinformatics analysis. JD, JC, SS, and AL designed and supervised the study. DF, AB, SS, and AL wrote the manuscript. All authors read and approved the manuscript.

### Conflict of interest statement

The authors declare that the research was conducted in the absence of any commercial or financial relationships that could be construed as a potential conflict of interest. The reviewer ES and handling Editor declared their shared affiliation.

## Supplementary material

Supplementary Worksheet 1 Differential expression analysis of *Flatr*-deficient Tregs. CD4^+^CD25^+^ Tregs from wildtype, Exon 1 KO, and Exon 2 KO mice were assessed by RNAseq (*n* = 3/group). Expression levels and statistical comparisons are given.

Supplementary Figure 1 *Flatr* deficient T cells show an IL2-independent delay in Treg induction *ex vivo*. Cultured naïve T cells (CD4^+^ CD62L^+^ CD44^−^) from spleen and lymph nodes of wildtype mice and Exon1 KO mice were activated with αCD3 and αCD28, with and without TGFβ at the indicated time points (*n* = 3). The assay was performed in the absence of IL-2. ^*^*P* < 0.05, ^**^*P* < 0.01, ^****^*P* < 0.0001 for WT vs. Exon1 KO at concentration of 0ng of TGFβ; #*P* < 0.05, ##*P* < 0.01, ###*P* < 0.001 for WT vs. Exon1 KO at concentration of 2.5 ng of TGFβ; §*P* < 0.05, §§*P* < 0.01 for WT vs. Exon1 KO at concentration of 5 ng of TGFβ. All data are means ± SEM.

Supplementary Figure 2 Global expression changes in *Flatr*-deficient Tregs. CD4^+^CD25^+^ Tregs from wildtype, Exon 1 KO, and Exon 2 KO mice were assessed by RNAseq (*n* = 3/group). PCA analysis displaying PC1 (17% of variance) and PC2 (16% of variance).

## References

[1] Rinn JL Chang HY. Genome regulation by long noncoding RNAs. Annu Rev Biochem. (2012) 81:145–66. 10.1146/annurev-biochem-051410-092902 22663078 PMC3858397

[2] Hu W Alvarez-Dominguez JR Lodish HF. Regulation of mammalian cell differentiation by long non-coding RNAs. EMBO Rep. (2012) 13:971–83. 10.1038/embor.2012.145 23070366 PMC3492712

[3] Mercer TR Dinger ME Mattick JS. Long non-coding RNAs: insights into functions. Nat Rev Genet. (2009) 10:155–9. 10.1038/nrg2521 19188922

[4] Kotzin JJ Spencer SP McCright SJ Kumar DBU Collet MA Mowel WK. The long non-coding RNA Morrbid regulates Bim and short-lived myeloid cell lifespan. Nature (2016) 537:239–43. 10.1038/nature19346 27525555 PMC5161578

[5] Wagner LA Christensen CJ Dunn DM Spangrude GJ Georgelas A Kelley L. EGO, a novel, noncoding RNA gene, regulates eosinophil granule protein transcript expression. Blood (2007) 109:5191–8. 10.1182/blood-2006-06-027987 17351112 PMC1890841

[6] Zhang X Lian Z Padden C Gerstein MB Rozowsky J Snyder M. A myelopoiesis-associated regulatory intergenic noncoding RNA transcript within the human HOXA cluster. Blood (2009) 113:2526–34. 10.1182/blood-2008-06-162164 19144990 PMC2656274

[7] Wang P Xue Y Han Y Lin L Wu C Xu S. The STAT3-binding long noncoding RNA lnc-DC controls human dendritic cell differentiation. TL - 344. Science (2014) 344 VN-:310–3. 10.1126/science.1251456 24744378

[8] Carpenter S Aiello D Atianand MK Ricci EP Gandhi P Hall LL. A long noncoding RNA mediates both activation and repression of immune response genes. Science (2013) 341:789–92. 10.1126/science.1240925 23907535 PMC4376668

[9] Li Z Chao T-C Chang K-Y Lin N Patil VS Shimizu C. The long noncoding RNA THRIL regulates TNF expression through its interaction with hnRNPL. Proc Natl Acad Sci. (2014) 111:1002–7. 10.1073/pnas.1313768111 24371310 PMC3903238

[10] Krawczyk M Emerson BM. P50-associated COX-2 Extragenic RNA (pacer) activates human COX-2 gene expression by occluding repressive NF-??B p50 complexes. Elife (2014) 2014:01776. 10.7554/eLife.01776 24843008 PMC4017649

[11] Imamura K Akimitsu N. Long non-coding RNAs involved in immune responses. Front Immunol. (2014) 5:e00573. 10.3389/fimmu.2014.00573 25431574 PMC4230175

[12] Isoda T Moore AJ He Z Chandra V Aida M Denholtz M. Non-coding transcription instructs chromatin folding and compartmentalization to dictate enhancer-promoter communication and T cell fate. Cell (2017) 171:103–19.e18. 10.1016/j.cell.2017.09.001 28938112 PMC5621651

[13] Gomez JA Wapinski OL Yang YW Bureau JF Gopinath S Monack DM. The NeST long ncRNA controls microbial susceptibility and epigenetic activation of the interferon-γ locus. Cell (2013) 152:743–54. 10.1016/j.cell.2013.01.015 23415224 PMC3577098

[14] Mattick JS. The genetic signatures of noncoding RNAs. PLoS Genet. (2009) 5:e1000459. 10.1371/journal.pgen.1000459 19390609 PMC2667263

[15] Benoist C Mathis D. Treg cells, life history, and diversity. Cold Spring Harb Perspect Biol (2012) 4:e007021. 10.1101/cshperspect.a007021 22952391 PMC3428763

[16] Buckner JH. Mechanisms of impaired regulation by CD4+CD25+FOXP3+ regulatory T cells in human autoimmune diseases. Nat Rev Immunol. (2010) 10:849–59. 10.1038/nri2889 21107346 PMC3046807

[17] Franceschini D Paroli M Francavilla V Videtta M Morrone S Labbadia G. PD-L1 negatively regulates CD4+CD25+Foxp3+ Tregs by limiting STAT-5 phosphorylation in patients chronically infected with HCV. J Clin Invest. (2009) 119:551–64. 10.1172/JCI36604 19229109 PMC2648671

[18] Dannull J Su Z Rizzieri D Yang BK Coleman D Yancey D. Enhancement of vaccine-mediated antitumor immunity in cancer patients after depletion of regulatory T cells. J Clin Invest. (2005) 115:3623–33. 10.1172/JCI25947 16308572 PMC1288834

[19] Hsieh C-S Lee H-M Lio C-WJ. Selection of regulatory T cells in the thymus. Nat Rev Immunol. (2012) 12:157–6. 10.1038/nri3155 22322317

[20] Atarashi K Tanoue T Oshima K Suda W Nagano Y Nishikawa H. Treg induction by a rationally selected mixture of Clostridia strains from the human microbiota. Nature (2013) 500:232–6. 10.1038/nature12331 23842501

[21] Zheng Y Josefowicz SZ Kas A Chu T-T Gavin MA Rudensky AY. Genome-wide analysis of Foxp3 target genes in developing and mature regulatory T cells. Nature (2007) 445:936–40. 10.1038/nature05563 17237761

[22] Brunkow ME Jeffery EW Hjerrild KA Paeper B Clark LB Yasayko S-A. Disruption of a new forkhead/winged-helix protein, scurfin, results in the fatal lymphoproliferative disorder of the scurfy mouse. Nat Genet. (2001) 27:68–73. 10.1038/83784 11138001

[23] Wildin RS Smyk-Pearson S Filipovich AH. Clinical and molecular features of the immunodysregulation, polyendocrinopathy, enteropathy, X linked (IPEX) syndrome. J Med Genet. (2002) 39:537–45. 10.1136/jmg.39.8.537 12161590 PMC1735203

[24] Jiang R Tang J Chen Y Deng L Ji J Xie Y. The long noncoding RNA lnc-EGFR stimulates T-regulatory cells differentiation thus promoting hepatocellular carcinoma immune evasion. Nat Commun. (2017) 8:15129. 10.1038/ncomms15129 28541302 PMC5529670

[25] Zemmour D Pratama A Loughhead SM Mathis D Benoist C. *Flicr*, a long noncoding RNA, modulates Foxp3 expression and autoimmunity. Proc Natl Acad Sci. (2017) 114:E3472–80. 10.1073/pnas.1700946114 28396406 PMC5410798

[26] Fontenot JD Rasmussen JP Williams LM Dooley JL Farr AG Rudensky AY. Regulatory T cell lineage specification by the forkhead transcription factor Foxp3. Immunity (2005) 22:329–41. 10.1016/j.immuni.2005.01.016 15780990

[27] Samstein RM Arvey A Josefowicz SZ Peng X Reynolds A Sandstrom R. Foxp3 exploits a pre-existent enhancer landscape for regulatory T cell lineage specification. Cell (2012) 151:153–66. 10.1016/j.cell.2012.06.053 23021222 PMC3493256

[28] van der Veeken J Gonzalez AJ Cho H Arvey A Hemmers S Leslie CS. Memory of Inflammation in Regulatory T Cells. Cell (2016) 166:977–90. 10.1016/j.cell.2016.07.006 27499023 PMC4996371

[29] Stubbington MJ Mahata B Svensson V Deonarine A Nissen JK Betz AG. An atlas of mouse CD4(+) T cell transcriptomes. Biol Direct. (2015) 10:14. 10.1186/s13062-015-0045-x 25886751 PMC4384382

[30] Pierson W Cauwe B Policheni A Schlenner SM Franckaert D Berges J. Antiapoptotic Mcl-1 is critical for the survival and niche-filling capacity of Foxp3+ regulatory T cells. Nat Immunol. (2013) 14:959–65. 10.1038/ni.2649 23852275 PMC4128388

[31] Campbell DJ Koch MA. Phenotypical and functional specialization of FOXP3+ regulatory T cells. Nat Rev Immunol. (2011) 11:119–30. 10.1038/nri2916 21267013 PMC3289970

[32] Li Z Li D Tsun A Li B. FOXP3(+) regulatory T cells and their functional regulation. Cell Mol Immunol. (2015) 2:1–8. 10.1038/cmi.2015.10 25683611 PMC4579651

[33] Rudra D deRoos P Chaudhry A Niec RE Arvey A Samstein RM. Transcription factor Foxp3 and its protein partners form a complex regulatory network. Nat Immunol. (2012) 13:1010–9. 10.1038/ni.2402 22922362 PMC3448012

[34] Kwon H-K Chen H-M Mathis D Benoist C. Different molecular complexes that mediate transcriptional induction and repression by FoxP3. Nat Immunol. (2017) 18:1238–48. 10.1038/ni.3835 28892470 PMC5679728

[35] Fu W Ergun A Lu T Hill JA Haxhinasto S Fassett MS. A multiply redundant genetic switch “locks in” the transcriptional signature of regulatory T cells. Nat Immunol. (2012) 13:972–80. 10.1038/ni.2420 22961053 PMC3698954

[36] Ohkura N Hamaguchi M Morikawa H Sugimura K Tanaka A Ito Y. T cell receptor stimulation-induced epigenetic changes and Foxp3 expression are independent and complementary events required for Treg cell development. Immunity (2012) 37:785–99. 10.1016/j.immuni.2012.09.010 23123060

[37] Kim H-P Leonard WJ. CREB/ATF-dependent T cell receptor-induced FoxP3 gene expression: a role for DNA methylation. J Exp Med. (2007) 204:1543–51. 10.1084/jem.20070109 17591856 PMC2118651

[38] Polansky JK Kretschmer K Freyer J Floess S Garbe A Baron U. DNA methylation controls Foxp3 gene expression. Eur J Immunol. (2008) 38:1654–63. 10.1002/eji.200838105 18493985

[39] Zheng Y Josefowicz S Chaudhry A Peng XP Forbush K Rudensky AY. Role of conserved non-coding DNA elements in the Foxp3 gene in regulatory T-cell fate. Nature (2010) 463:808–12. 10.1038/Nature08750 20072126 PMC2884187

[40] Delacher M Imbusch CD Weichenhan D Breiling A Hotz-Wagenblatt A Träger U. Genome-wide DNA-methylation landscape defines specialization of regulatory T cells in tissues. Nat Immunol. (2017) 18:1160–72. 10.1038/ni.3799 28783152 PMC5912503

[41] Cobb BS Hertweck A Smith J O'Connor E Graf D Cook T. A role for Dicer in immune regulation. J Exp Med. (2006) 203:2519–27. 10.1084/jem.20061692 17060477 PMC2118134

[42] Zhou X Jeker LT Fife BT Zhu S Anderson MS McManus MT. Selective miRNA disruption in T reg cells leads to uncontrolled autoimmunity. J Exp Med. (2008) 205:1983–91. 10.1084/jem.20080707 18725525 PMC2526194

[43] Chong MMW Rasmussen JP Rudensky AY Littman DR. The RNAseIII enzyme Drosha is critical in T cells for preventing lethal inflammatory disease. J Exp Med. (2008) 205:2005–17. 10.1084/jem.20081219 18725527 PMC2526196

[44] Liston A Lu L-F O'Carroll D Tarakhovsky A Rudensky AY. Dicer-dependent microRNA pathway safeguards regulatory T cell function. J Exp Med. (2008) 205:1993–2004. 10.1084/jem.20081062 18725526 PMC2526195

[45] Lu LF Boldin MP Chaudhry A Lin LL Taganov KD Hanada T. Function of miR-146a in controlling Treg cell-mediated regulation of Th1 responses. Cell (2010) 142:914–29. 10.1016/j.cell.2010.08.012 20850013 PMC3049116

[46] Lu LF Thai TH Calado DP Chaudhry A Kubo M Tanaka K. Foxp3-dependent microRNA155 confers competitive fitness to regulatory T cells by targeting SOCS1 protein. Immunity (2009) 30:80–91. 10.1016/j.immuni.2008.11.010 19144316 PMC2654249

[47] Yang HY Barbi J Wu CY Zheng Y Vignali PDA Wu X. MicroRNA-17 modulates regulatory T cell function by targeting co-regulators of the Foxp3 transcription factor. Immunity (2016) 45:83–93. 10.1016/j.immuni.2016.06.022 27438767 PMC4957244

[48] Li JQ Hu SY Wang ZY Lin J Jian S Dong YC. Long non-coding RNA MEG3 inhibits microRNA-125a-5p expression and induces immune imbalance of Treg/Th17 in immune thrombocytopenic purpura. Biomed Pharmacother. (2016) 83:905–11. 10.1016/j.biopha.2016.07.057 27522004

[49] Qiao YQ Huang ML Xu AT Zhao D Ran ZH Shen J. LncRNA DQ786243 affects Treg related CREB and Foxp3 expression in Crohn's disease. J Biomed Sci. (2013) 20:87. 10.1186/1423-0127-20-87 24289115 PMC4174896

[50] Hill JA Feuerer M Tash K Haxhinasto S Perez J Melamed R. Foxp3 transcription-factor-dependent and -independent regulation of the regulatory T cell transcriptional signature. Immunity (2007) 27:786–800. 10.1016/j.immuni.2007.09.010 18024188

[51] Gavin MA Rasmussen JP Fontenot JD Vasta V Manganiello VC Beavo JA. Foxp3-dependent programme of regulatory T-cell differentiation. Nature (2007) 445:771–5. 10.1038/nature05543 17220874

[52] Schlenner SM Weigmann B Ruan Q Chen Y von Boehmer H. Smad3 binding to the foxp3 enhancer is dispensable for the development of regulatory T cells with the exception of the gut. J Exp Med. (2012) 209:1529–35. 10.1084/jem.20112646 22908322 PMC3428940

[53] Feng Y Arvey A Chinen T Van Der Veeken J Gasteiger G Rudensky AY. Control of the inheritance of regulatory T cell identity by a cis element in the foxp3 locus. Cell (2014) 158:749–63. 10.1016/j.cell.2014.07.031 25126783 PMC4151558

[54] Li X Liang Y Leblanc M Benner C Zheng Y. Function of a foxp3 cis-element in protecting regulatory T cell identity. Cell (2014) 158:734–48. 10.1016/j.cell.2014.07.030 25126782 PMC4151505

[55] Ogawa C Tone Y Tsuda M Peter C Waldmann H Tone M. TGF–mediated Foxp3 gene expression is cooperatively regulated by Stat5, Creb, and AP-1 through CNS2. J Immunol. (2014) 192:475–83. 10.4049/jimmunol.1301892 24298014 PMC3905572

[56] Liston A Nutsch KM Farr AG Lund JM Rasmussen JP Koni PA. Differentiation of regulatory Foxp3+ T cells in the thymic cortex. Proc Natl Acad Sci USA. (2008) 105:11903–8. 10.1073/pnas.0801506105 18695219 PMC2575273

[57] Hu G Tang Q Sharma S Yu F Escobar TM Muljo SA. Expression and regulation of intergenic long noncoding RNAs during T cell development and differentiation. Nat Immunol. (2013) 14:1190–8. 10.1038/ni.2712 24056746 PMC3805781

[58] Morikawa H Ohkura N Vandenbon A Itoh M Nagao-Sato S Kawaji H. Differential roles of epigenetic changes and Foxp3 expression in regulatory T cell-specific transcriptional regulation. Proc Natl Acad Sci USA. (2014) 111:5289–94. 10.1073/pnas.1312717110 24706905 PMC3986152

[59] Ovcharenko I Nobrega MA Loots GG Stubbs L. ECR Browser: a tool for visualizing and accessing data from comparisons of multiple vertebrate genomes. Nucleic Acids Res. (2004) 32:W280–6. 10.1093/nar/gkh355 15215395 PMC441493

