# Supplementary figures and images for: The Long Non-coding RNA Flatr Anticipates Foxp3 Expression in Regulatory T Cells
Source: Front Immunol. 2018 Sep 25;9:1989. doi: 10.3389/fimmu.2018.01989 (PMC6167443; doi:10.3389/fimmu.2018.01989)

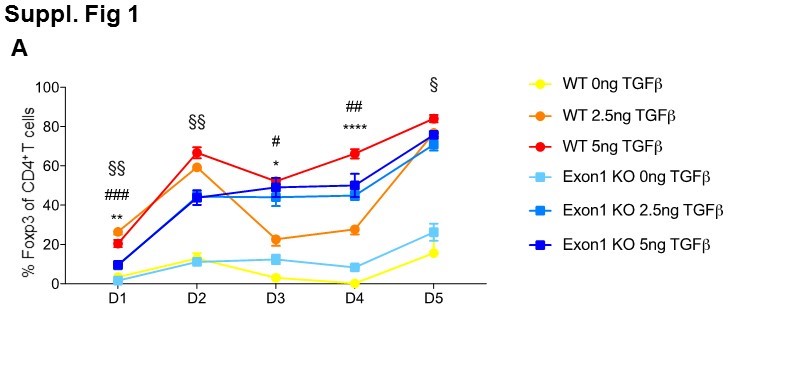

Supplement: Supplementary Figure 1 — Flatr deficient T cells show an IL2-independent delay in Treg induction ex vivo. Cultured naïve T cells (CD4+ CD62L+ CD44−) from spleen and lymph nodes of wildtype mice and Exon1 KO mice were activated with αCD3 and αCD28, with and without TGFβ at the indicated time points (n = 3). The assay was performed in the absence of IL-2. *P < 0.05, **P < 0.01, ****P < 0.0001 for WT vs. Exon1 KO at concentration of 0ng of TGFβ; #P < 0.05, ##P < 0.01, ###P < 0.001 for WT vs. Exon1 KO at concentration of 2.5 ng of TGFβ; §P < 0.05, §§P < 0.01 for WT vs. Exon1 KO at concentration of 5 ng of TGFβ. All data are means ± SEM. [file Image_1.JPEG]

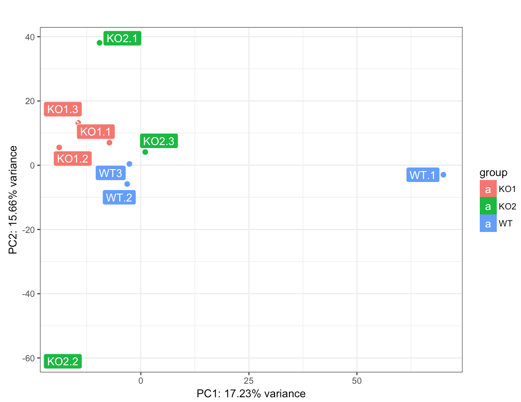

Supplement: Supplementary Figure 2 — Global expression changes in Flatr-deficient Tregs. CD4+CD25+ Tregs from wildtype, Exon 1 KO, and Exon 2 KO mice were assessed by RNAseq (n = 3/group). PCA analysis displaying PC1 (17% of variance) and PC2 (16% of variance). [file Image_2.png]
